# The impact of psychological interventions on anxiety and depression in cancer pain patients: A meta-analysis

**DOI:** 10.1097/MD.0000000000048585

**Published:** 2026-05-08

**Authors:** Xiao Pu Wu, Shao Chu Zheng, Yu Ting Huang, Yi Wang, Yue Wu, Shi Hao Yang, Shang Li, Jin Ling Tang, Cao Qing, Yun Jiang, Wei Lu, Chong Xi Bao, Qin Zhe Zhang, Jing Luo, Jin Liang Kong

**Affiliations:** a Department of Pulmonary and Critical Care Medicine, The First Affiliated Hospital of Guangxi Medical University, Nanning, Guangxi, China; b Department of Ophthalmology, The Tenth Affiliated Hospital of Guangxi Medical University, Qinzhou, Guangxi, China; c Department of Endocrinology and Metabolism Nephrology, Guangxi Medical University Cancer Center, Nanning, Guangxi, China.

**Keywords:** anxiety, cancer pain, depression, meta-analysis, negative affect, psychological intervention

## Abstract

**Background:**

Cancer pain is frequently accompanied by anxiety and depression, which severely impair patients’ quality of life. Although psychological interventions are widely applied in clinical practice, their overall effect on alleviating anxiety and depression among cancer pain patients remains to be systematically evaluated.

**Methods:**

This meta-analysis strictly followed the Preferred Reporting Items for Systematic Reviews and Meta-Analyses 2020 guidelines. Randomized controlled trials of psychological intervention for anxiety and depression in cancer pain patients were searched across PubMed, Web of Science, Embase, the Cochrane Library, Wanfang, China national knowledge infrastructure, VIP, and China Biomedical Literature Database from inception to May 21, 2025. According to the Cochrane systematic assessment method, literature screening, quality evaluation, and information extraction were carried out by 2 independent reviewers. Meta-analysis was conducted with RevMan 5.3 software, including heterogeneity test, effect size pooling, sensitivity analysis, subgroup analysis, and publication bias assessment.

**Results:**

A total of 11 randomized controlled trials involving 879 patients (440 in the intervention group and 439 in the control group) were included. Pooled analysis showed that psychological interventions significantly reduced anxiety scores (weighted mean difference = −8.82, 95% confidence interval [−10.52, −7.12], *P* < .001) and depression scores (weighted mean difference = −9.39, 95% confidence interval [−11.79, −6.98], *P* < .001) in cancer pain patients compared with conventional care. Sensitivity analysis confirmed the robustness of the results, and prespecified subgroup analyses (by intervention duration, mean age, sex ratio) failed to identify significant sources of heterogeneity. Funnel plots combined with Begg and Egger tests indicated no significant publication bias.

**Conclusion:**

Psychological intervention can effectively improve the anxiety and depression of cancer patients during chemotherapy, and future studies need to pay attention to the impact of factors such as the type of cancer, cancer pain, patient population, and specific psychological interventions.

## 1. Introduction

Cancer pain refers to pain caused by primary tumors, tumor metastasis, or antitumor therapy and is one of the most common and distressing symptoms in cancer patients.^[1,2]^ According to statistics, about 30% to 50% of cancer patients and 66% of advanced cancer patients experience cancer pain.^[3,4]^ Based on the biopsychosocial model, cancer pain is not a simple physiological symptom but a complex clinical phenomenon shaped by the interaction of biological, psychological, and social factors.^[5]^ This pain not only causes physical pain but also seriously affects the patient’s psychological state, social functioning, and overall quality of life.^[6]^ Moreover, cancer pain patients are often accompanied by negative emotions such as anxiety, depression, fear, stigma, nervousness, and despair, which further aggravate pain perception and form a vicious cycle, thereby affecting patient compliance and resulting in poor tumor treatment outcomes.^[7]^

At present, the current treatment methods for cancer pain are mainly the “three-step” drug pain relief method recommended by the World Health Organization, which has the advantages of fast onset and rapid action, but there is dependence on large doses and long-term applications, and at the same time it will cause certain adverse reactions, and the degree of pain relief in some patients is not ideal.^[8]^ As an important nonpharmacological treatment method, psychological intervention (including cognitive behavioral therapy [CBT], mindfulness-based stress reduction, social support therapy, and traditional Chinese medicine emotional adjustment) can regulate patients’ cognitive appraisal of pain, modulate neuroendocrine and immune responses, and enhance social support, thereby effectively reducing pain intensity, improving negative emotions, and increasing treatment compliance.^[9,10]^

In recent years, several systematic reviews and meta-analyses have explored the role of psychological interventions in cancer care.^[11]^ However, most existing studies have focused on the general cancer population rather than specifically targeting cancer pain patients – a subgroup with more severe negative emotions and closer pain-emotion interaction. Furthermore, the current evidence on the efficacy of psychological interventions for negative emotions in cancer pain patients remains inconsistent, and the optimal intervention type, duration, and applicable population have not been clarified. Based on the biopsychosocial model of cancer pain, this meta-analysis aims to systematically synthesize high-quality randomized controlled trials (RCTs) on psychological interventions for anxiety and depression in cancer pain patients, evaluate the overall efficacy of psychological interventions, explore potential heterogeneity sources, and provide more targeted and reliable evidence for optimizing the biopsychosocial comprehensive management of cancer pain and improving the quality of life of cancer pain patients.

## 2. Methods

This meta-analysis strictly followed the guidelines of the Preferred Reporting Items for Systematic Reviews and Meta-Analyses 2020, and the overall research design was based on the biopsychosocial model of cancer pain and the PICO (Population, Intervention, Comparison, Outcome) principle to ensure methodological rigor, transparency, and reproducibility.^[12]^

### 2.1. Ethical statement

As a secondary synthesis of previously published data, all included primary studies had obtained their own necessary ethical approvals and informed consent from participants. Therefore, this meta-analysis did not require separate ethical approval from an ethics committee or institutional review board.

### 2.2. Search strategy

#### 2.2.1. Search databases and time range

We searched international electronic databases (PubMed, Web of Science, Embase, the Cochrane Library) and Chinese electronic databases (China national knowledge infrastructure, VIP, Wanfang, China Biomedical Literature Database) from their inception to May 21, 2025. No manual retrieval or gray literature search was performed due to the high comprehensiveness of the above electronic databases.

#### 2.2.2. Search terms and combination logic

The search strategy combined MeSH terms (subject headings) and free words, with core search terms divided into 3 modules: cancer pain, negative emotions (anxiety/depression), and psychological intervention. The filter term “randomized controlled trial” was added to restrict study type. The specific search terms were as follows: (“Cancer Pain” OR “cancer pain patients” OR “Neoplastic pain” OR “Cancer-Related Pain” OR “Anxiety” OR “Depression” OR “Depressive Symptoms” OR “negative emotion” OR “unhealthy emotions”) and (“Psychosocial Intervention” OR “Intervention Psychosocial” OR “Psychological Intervention Treatment” OR “Psychological Intervention methods”), and (“randomized controlled trial” OR “randomized controlled study”). The detailed search strategy for each database is presented in Supplemental Text S1, Supplemental Digital Content.

#### 2.2.3. Supplementary retrieval

The reference lists of included studies, relevant systematic reviews, and meta-analyses were manually checked to identify additional potentially eligible studies and avoid missing relevant literature.

### 2.3. Inclusion and exclusion criteria

#### 2.3.1. Inclusion criteria

The inclusion criteria were as follows: RCT; research subjects: patients diagnosed with malignant tumors and cancer-related pain by pathological examination; intervention measures: the experimental group adopts a series of psychological intervention measures or combined with conventional treatment, and the control group adopts conventional treatment intervention; and outcome indicators: Self-rating Anxiety Scale (SAS) and Self-rating Depression Scale (SDS).

#### 2.3.2. Exclusion criteria

The exclusion criteria were as follows: the full text is not available; unable to obtain valid data; systematic reviews, reviews, and non-RCTs; and duplicate publications from the same research team.

### 2.4. Study selection and data extraction

Initial screening and rescreening of the retrieved literature using EndNote 20 (Clarivate Analytics, Philadelphia) were independently carried out by 2 evidence-based trained investigators. The screening content included study type, research content, research subjects, and outcome indicators. Relevant data were extracted, and a table of basic characteristics of the literature was prepared, including authors, publication years, gender ratio, mean age, sample size (intervention group/control group), intervention measures, intervention duration, and outcome indicators (SAS/SDS scores before and after intervention). If the 2 investigators had different opinions and could not reach a consensus through discussion, they consulted a third analyst for resolution.

### 2.5. Quality assessment

The quality of RCTs was assessed using the Cochrane risk of bias assessment tool,^[13]^ including randomized methods, allocation concealment, blinding, data completeness, selective reporting of study results, and other biases. Each domain was rated as low risk, unclear risk, or high risk.

### 2.6. Statistical analysis

Meta-analysis was performed using RevMan 5.3 software (Cochrane Collaboration, London, UK). Heterogeneity test: The *Q* test method (χ^2^ value) was used to determine whether there is heterogeneity between studies. When *P* > .1 and *I*^2^ < 50%, which means that the statistical heterogeneity between studies was small, and the fixed-effect model was used to analyze it. When *P* < .1 and *I*^2^ ≥ 50%, indicating that there is large statistical heterogeneity between studies, a random-effects model is used, and sensitivity analysis or subgroup analysis is used to explore the source of heterogeneity between studies. The test level is α = 0.05; if the source of heterogeneity cannot be determined, descriptive analysis is used. Effect size pooling: All outcome indicators in this study were continuous data; since the same measurement tools (SAS/SDS) were used in all included studies, the weighted mean difference (MD) with 95% confidence interval (CI) was used to express the effect size. Publication bias assessment: Funnel plots and Begg/Egger tests were used to assess publication bias.

## 3. Results

### 3.1. Study selection

**Figure 1.**
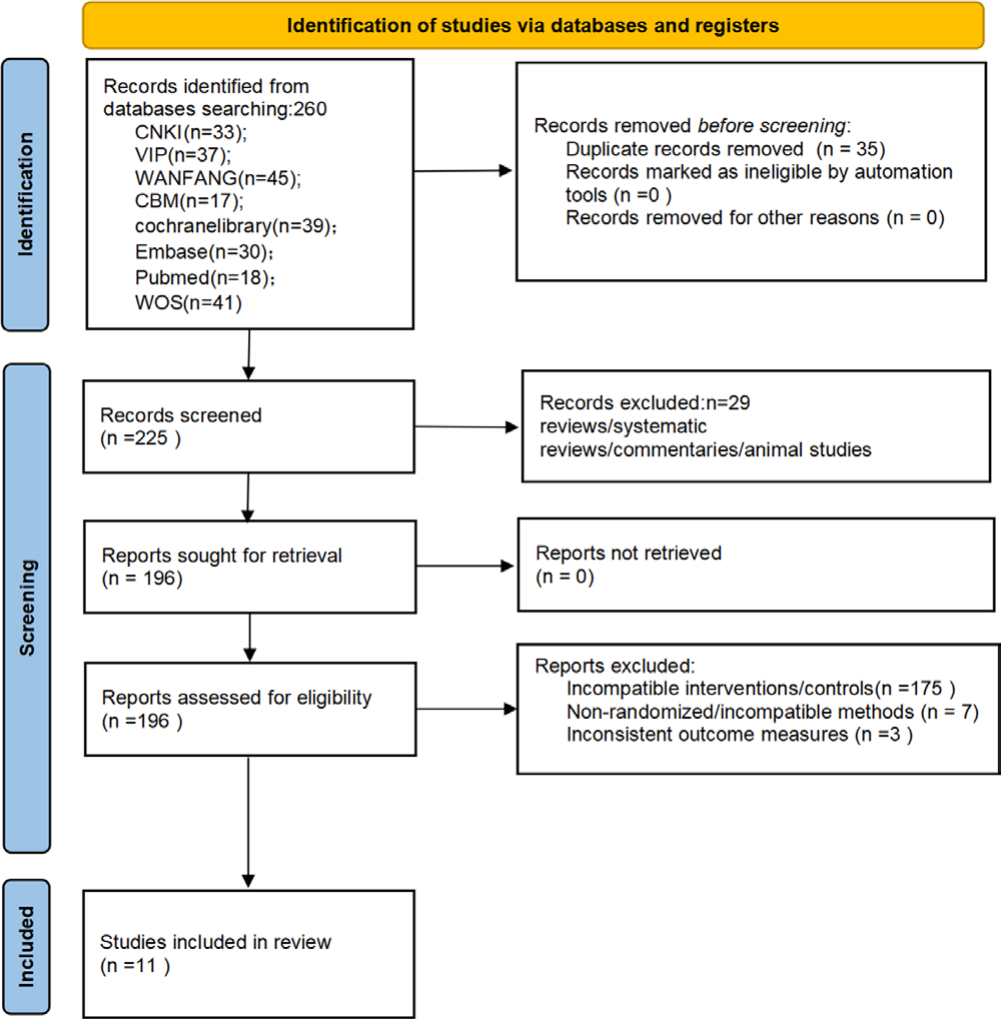
Flowchart of studies screening. CBM = China Biomedical Literature Database, CNKI = China national knowledge infrastructure, WOS = web of science.

A total of 260 records were initially retrieved from the 8 electronic databases. After removing 35 duplicate records, 225 unique records were obtained. Through title and abstract screening, 29 records with ineligible publication types and 175 irrelevant records were excluded, leaving 21 full-text articles for further evaluation. After full-text reading, 7 articles were excluded for not being RCTs or for having serious methodological flaws, and 3 articles were excluded for inconsistent outcome indicators or unavailable valid data. Finally, 11 RCTs involving 879 patients were included in this meta-analysis.^[14–24]^ The detailed study selection process is shown in Figure 1.

### 3.2. Study characteristics and quality

**Figure 2.**
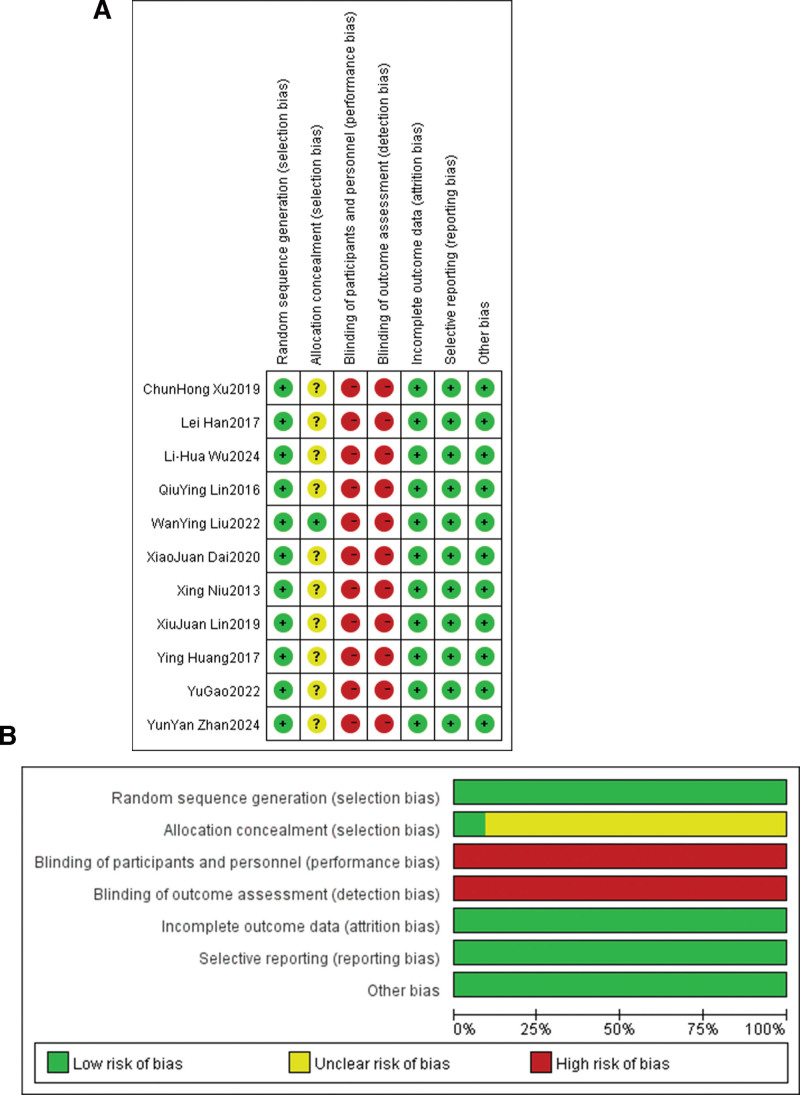
Risk assessment of bias in the included literature. (A) Risk of bias for each study; (B) overall risk of bias proportions. Green indicates low risk, yellow indicates unclear risk, and red indicates high risk.

The 11 included RCTs enrolled 879 patients (intervention: 440; control: 439). All studies assessed anxiety using SAS and depression using SDS. Interventions varied: psychological nursing, general psychological intervention, CBT-based interventions, narrative nursing, traditional Chinese medicine emotional adjustment, mindfulness-based stress reduction, and mental health education combined with pain education. Control groups received usual care (e.g., conventional nursing, pharmacological analgesia). Intervention durations ranged from 2 to 12 weeks. Detailed characteristics are presented in Table 1. The Cochrane risk of bias assessment showed that the overall quality of the included studies was moderate: most studies clearly reported the random sequence generation method (low risk), but the majority of studies did not report allocation concealment and blinding methods (unclear risk). All studies had complete outcome data (low risk), and no selective reporting or other obvious biases were found. The specific risk of bias distribution is shown in Figure 2.

**Table 1:** Characteristics of the included studies.

Study	Test method	Sample size	Sex ratio	Average age		Intervention measure		Experimental period	Outcome measure
		T/C		T	C	T	C		
Xu^[14]^	RCT, OL	40/40	130%	58.62 ± 4.51	58.42 ± 4.63	① + ②	②	Not mentioned	SAS, SDS
Han et al^[15]^	RCT, OL	43/42	142%	Not mentioned	Not mentioned	③ + ④	④	2 wk	SAS, SDS
Wu et al^[16]^	RCT, OL	40/40	105%	64.26 ± 6.29	64.13 ± 6.26	⑤	②	Not mentioned	SAS, SDS
Lin et al^[17]^	RCT, OL	26/26	330%	52.92 ± 0.46	54.46 ± 10.61	①	②	Not mentioned	SAS, SDS
Liu^[18]^	RCT, OL	37/38	120%	48.41 ± 7.80	50.71 ± 7.12	⑥ + ②	②	5 wk	SAS, SDS
Dai^[19]^	RCT, OL	34/34	106%	Not mentioned	Not mentioned	① + ②	②	Not mentioned	SAS, SDS
Niu^[20]^	RCT, OL	35/35	105%	Not mentioned	Not mentioned	⑦ + ⑧ + ⑨	⑧ + ⑨	4 wk	SAS
Lin^[21]^	RCT, OL	45/45	100%	50.1 ± 10.23	52.4 ± 10.42	⑩ + ⑪ + ⑫	⑪ + ⑫	7 wk	SAS, SDS
Huang et al^[22]^	RCT, OL	58/58	164%	60 ± 12.36	62 ± 11.52	③ + ⑬ + ②	⑬ + ②	6–8 wk	SAS, SDS
Gao et al^[23]^	RCT, OL	34/33	109%	56.05 ± 6.81	55.71 ± 6.72	① + ⑭	⑭	2 wk	SAS, SDS
Zhan et al^[24]^	RCT, OL	48/48	109%	57.75 ± 8.69	58.23 ± 8.18	① + ④ + ②	④ + ②	2 wk	SAS, SDS

Sex ratio = (number of males ÷ number of females) × 100%.

① = psychological nursing intervention, ② = conventional nursing, ③ = psychological intervention, ④ = 3-step analgesia, ⑤ = narrative nursing, ⑥ = emotional adjustment program of traditional Chinese medicine, ⑦ = psychological behavioral interventions, ⑧ = routine pain care, ⑨ = pharmacological analgesia, ⑩ = mindfulness-based stress reduction, ⑪ = mental health education + pain education, ⑫ = pain education, ⑬ = pharmacotherapy, ⑭ = oxycodone hydrochloride sustained-release tablets, C = control group, OL = open-label, RCT = randomized controlled trial, SAS = Self-rating Anxiety Scale, SDS = Self-rating Depression Scale, T = treatment group.

### 3.3. Effect of psychological intervention on anxiety state (SAS) in cancer pain patients

**Figure 3.**
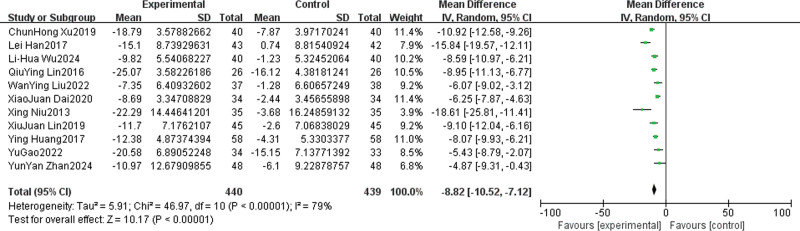
Forest diagram of the effect of psychological intervention on anxiety state. CI = confidence interval, IV = intravenous, SD = standard deviation.

All 11 included studies reported SAS scores before and after intervention.^[14–24]^ Heterogeneity test showed significant statistical heterogeneity between studies (*I*^2^ = 79%, *P* < .001); therefore, the random-effects model was used for pooled analysis. The results showed that the post-intervention SAS score in the psychological intervention group was significantly lower than that in the control group (MD = −8.82, 95% CI [−10.52, −7.12], *P* < .001; Fig. 3), indicating that psychological intervention can effectively reduce anxiety in cancer pain patients.

### 3.4. Effects of psychological interventions on depressive status (SDS) in cancer pain patients

**Figure 4.**
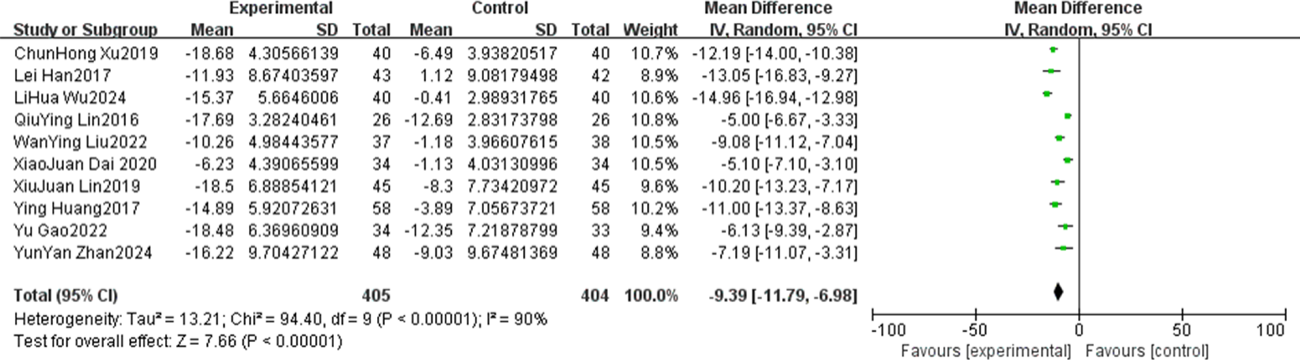
Forest plot of the effect of psychological intervention on depressive state. CI = confidence interval, IV = intravenous, SD = standard deviation.

Ten of the 11 included studies reported SDS scores before and after intervention.^[14–19,21–24]^ Heterogeneity test showed substantial statistical heterogeneity between studies (*I*^2^ = 90%, *P* < .001); therefore, the random-effects model was used for pooled analysis. The results showed that the post-intervention SDS score in the psychological intervention group was significantly lower than that in the control group (MD = −9.39, 95% CI [−11.79, −6.98], *P* < .001; Fig. 4), indicating that psychological intervention can effectively reduce depression in cancer pain patients.

### 3.5. Sensitivity and subgroup analysis results

#### 3.5.1. Sensitivity analysis

Sensitivity analysis was performed by sequentially removing each included study and recalculating the pooled effect size for anxiety and depression. The results showed that the pooled MD values and 95% CIs for SAS and SDS scores did not change significantly, and the heterogeneity did not decrease substantially after removing any single study. This indicates that the results of this meta-analysis are robust and not affected by a single study.

#### 3.5.2. Subgroup analysis

Prespecified subgroup analyses were conducted based on intervention duration, mean age, and female proportion to explore the source of heterogeneity. The results showed that there was still significant heterogeneity in each subgroup, and no significant difference in intervention efficacy was found between different subgroups (Table 2). This suggests that the effect of psychological intervention on improving anxiety and depression in cancer pain patients is consistent across different intervention durations, age groups, and gender ratios, and these factors are not the main sources of heterogeneity in this study.

**Table 2:** Subgroup analysis of the effects of psychological intervention on anxiety and depression in cancer pain patients.

Items	Subgroup	SAS					SDS				
		Study	Effect model	MD (95% CI)	*P*	*I*^2^ (%)	Study	Effect model	MD (95% CI)	*P*	*I*^2^ (%)
Period	≤4 wk	4^[15,20,23,24]^	①	−10.85 (−17.40, −4.30)	<.1	89	3^[15,23,24]^	①	−8.73 (−12.94, −4.53)	<.1	75
	>4 wk	3^[18,21,22]^	②	−7.86 (−9.24, −6.47)	>.1	7	3^[18,21,22]^	②	−9.96 (−11.34, −8.58)	>.1	0
	Combine	7	①	−9.12 (−11.98, −6.26)	<.1	81	6	①	−9.48 (−11.24, −7.72)	>.05	54
Average age	>55 yr	5^[14,16,22–24]^	①	−8.06 (−10.11, −6.01)	<.1	71	5^[14,16,22–24]^	①	−10.58 (−13.48, −7.68)	<.1	85
	≤55 yr	3^[17,18,21]^	②	−8.24 (−9.74, −6.73)	>.1	29	3^[17,18,21]^	①	−7.95 (−11.24, −4.65)	<.1	85
	Combine	8	①	−8.17 (−9.56, −6.79)	<.05	90	8	①	−9.55 (−12.17, −6.93)	<.1	90
Sex ratio	≥130%	4^[14,15,17,22]^	①	−10.55 (−13.05, −8.06)	<.1	81	4^[14,15,17,22]^	①	−10.19 (−14.27, −6.10)	<.1	93
	<130%	7^[16,18–21,23,24]^	①	−7.50 (−9.40, −5.59)	<.1	64	6^[16,18,19,21,23,24]^	①	−8.84 (−12.20, −5.49)	<.1	91
	Combine	11	①	−8.82 (−10.52, −7.12)	<.1	79	10	①	−9.39 (−11.79, −6.98)	<.1	90

① = random-effects model, ② = fixed-effects model, CI = confidence interval, MD = weighted mean difference, SAS = Self-rating Anxiety Scale, SDS = Self-rating Depression Scale.

### 3.6. Publication bias analysis

**Figure 5.**
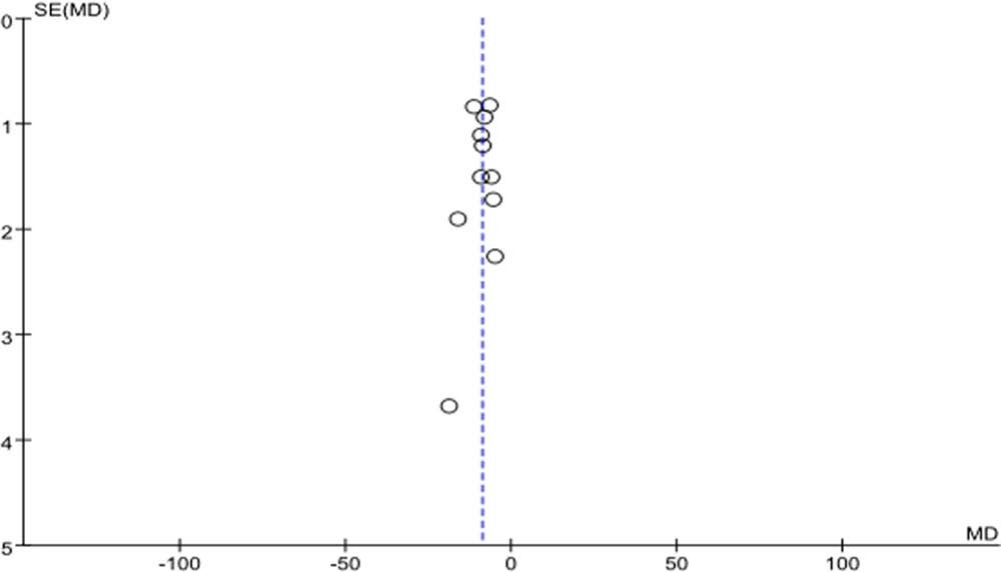
SAS funnel diagram. MD = weighted mean difference, SAS = Self-rating Anxiety Scale, SE = standard error.

**Figure 6.**
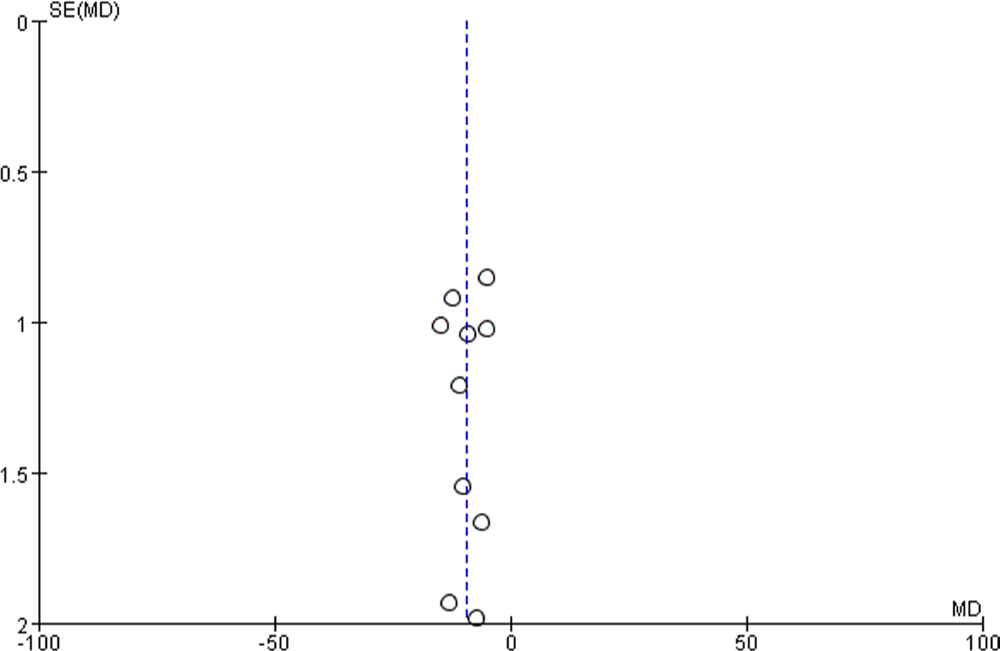
SDS funnel diagram. MD = weighted mean difference, SDS = Self-rating Depression Scale, SE = standard error.

Funnel plots for SAS and SDS scores were drawn to visually assess publication bias, and the results showed that the points in the funnel plots were basically symmetrical (Figs. 5 and 6), with no obvious publication bias. Quantitative statistical assessment by the Begg test and Egger test further confirmed this result (SAS: Begg *P* = .76, Egger *P* = .49; SDS: Begg *P* = 1.00, Egger *P* = .81, all *P* > .05). This indicated that there was no significant publication bias in this meta-analysis.

## 4. Discussion

Cancer pain is one of the most common symptoms in cancer patients, generally referring to pain caused by the cancer itself, related pathological lesions, or anticancer therapy, and it is mostly chronic in nature. It not only inflicts physical suffering on patients but also triggers severe negative emotions, among which anxiety and depression are the most prevalent.^[25,26]^ Anxiety and depression not only impair patients’ quality of life but also reduce their adherence to tumor treatment regimens and erode their will to survive. In turn, patients with cancer pain often experience helplessness and fear due to the persistence and unpredictability of pain, which further exacerbates their anxiety and depressive symptoms.^[27]^ Cancer pain is a complex phenomenon involving biological, psychological, and social factors, and the mechanisms underlying its interaction with negative emotional disorders such as anxiety and depression can be analyzed from 2 perspectives. Biologically,^[28,29]^ chronic cancer pain continuously activates the hypothalamic-pituitary-adrenal axis, leading to an abnormal elevation of stress hormones such as cortisol. This elevation inhibits the function of the prefrontal cortex – the core brain region for emotional regulation – enhances amygdalar reactivity, disrupts the neural balance of emotional regulation, and directly induces or exacerbates anxiety and depression. Functional MRI studies confirmed that overactivation of pain-related brain regions (insula, anterior cingulate gyrus, and other regions ) was positively correlated with the degree of anxiety and depression, while the anterior cingulate gyrus and other brain regions with a high distribution of glucocorticoid receptors were associated with increased neuronal deletion and increased adrenal cortisol.^[30,31]^ From the psychosocial level, pain is often associated with a series of adverse symptoms such as anxiety and depression due to psychosocial stress, and in long-term anxiety and depression, the patient’s immune system will also be suppressed, resulting in a decrease in immune function, which is not conducive to recovery.^[32]^ In addition, anxiety and depression may also affect patients’ adherence to treatment, making them resistant to treatment or even abandoning treatment, thus forming a vicious cycle of “pain → negative emotions → exacerbated pain.”

Based on the biopsychosocial model, psychological interventions alleviate anxiety and depression in patients with cancer pain through multi-dimensional targets, with core mechanisms involving cognitive-behavioral regulation, neuroendocrine modulation, and enhanced social support.^[33–35]^ Common psychological intervention methods and their corresponding mechanisms are summarized as follows: CBT: This therapy helps patients establish positive coping strategies by changing their cognitive and behavioral patterns about cancer pain.^[36]^ This method can help patients recognize the irrationality of their own cognition and guide them to pay attention to the controllability of pain, such as pain relief through medication, physical therapy, and other methods. At the same time, patients can also learn some relaxation techniques, such as deep breathing and progressive muscle relaxation, to reduce physical tension and anxiety. Mindfulness meditation: Mindfulness meditation is a training method that develops awareness and acceptance by focusing on the present experience. Cancer pain patients can focus on their breath or body sensations through mindfulness meditation practices, reducing excessive attention and worry about pain.^[37]^ This method can help patients relieve anxiety and depression, improve mental toughness, improve sleep quality, and reduce pain perception. Music therapy^[38]^: Music has a powerful emotionally regulating effect, which can promote the secretion of dopamine and endorphins in the brain, making patients emotionally relaxed. Cancer pain patients can choose some soothing music, such as classical music and nature sounds, to distract from pain and relieve anxiety and depression in the process of listening to music. Social support therapy^[39,40]^: Cancer patients often feel lonely and helpless, lacking social support. Social support therapy can help patients establish a good social support system, such as maintaining close communication with family, friends, and medical staff, sharing their feelings and experiences, or joining a cancer patient support group to support and encourage each other with patients who are sympathetic to each other, face diseases and pain together, and ultimately achieve the effect of enhancing patients’ psychological resilience and relieving anxiety and depression.

The results of this meta-analysis demonstrated that psychological interventions significantly reduced scores on the SAS and SDS in patients with cancer pain, with clinically significant efficacy (MD = −8.82 for anxiety, MD = −9.39 for depression). These findings are consistent with those of previous relevant studies,^[41,42]^ further confirming the value of psychological interventions in improving negative emotions in patients with cancer pain. However, compared with prior research, this meta-analysis has the following advantages: a specific study population: focusing on patients with cancer pain – a subgroup with a closer pain-emotion interaction – yielding more targeted results; up-to-date research data: including studies published up to 2025, reflecting the latest research progress; and rigorous methodological design: conducting rigorous subgroup and sensitivity analyses based on the biopsychosocial model, resulting in more robust findings.

Based on the biopsychosocial model of cancer and relevant clinical practice guidelines,^[43]^ the results of this meta-analysis provide important clinical implications for the comprehensive management of cancer pain. First, psychological interventions should be integrated into routine comprehensive cancer pain treatment as a safe and effective nonpharmacological treatment; psychological interventions should be combined with pharmacologic analgesia to form a 3-dimensional “biopsychosocial” comprehensive treatment system for cancer pain. Second, personalized psychological interventions should be implemented. Appropriate intervention types and durations should be selected based on patients’ tumor type, pain severity, age, educational level, and psychological state (e.g., long-term interventions for patients with severe cancer pain). Third, a multidisciplinary collaboration model should be established: in clinical practice, the effective implementation of psychological interventions requires close collaboration among a multidisciplinary team, including physicians, nurses, psychologists, and social workers. During treatment, physicians and nurses should monitor patients’ psychological status, promptly detect anxiety and depression in cancer patients, and provide corresponding psychological support and guidance. Psychologists can develop personalized psychological intervention plans for patients with cancer pain through professional psychological assessment and treatment. Social workers can provide cancer patients with social resources and support to help them address daily life difficulties.

Significant heterogeneity was observed in this meta-analysis (*I*^2^ = 79% for anxiety, *I*^2^ = 90% for depression). This is a common occurrence in meta-analyses of psychological interventions. Based on the biopsychosocial model, potential sources of heterogeneity in this study may include: in terms of research design, although the general flow of psychological intervention is consistent, the specific implementation process and specific psychological intervention methods are not completely unified; as far as the research subjects are concerned, the 11 articles included in this study are all cancer pain patients, and each study or different studies may contain cancer pain caused by multiple malignant tumors, and the effects of bad emotions caused by different tumors may be inconsistent, causing inconsistencies in the effects of intervention and treatment. The differences in age, psychological intervention time, gender, and other factors among patients will also affect their cognition. In terms of outcome measures, the included studies used patient self-assessment tools (SAS scale and SDS scale) to assess the severity of adverse moods, and different patients may feel differently about the specific scoring items of the self-rating scale, and the self-score may have large deviations. Regional and cultural differences (social dimension): all included studies were conducted in Asian populations (predominantly Chinese), and variations in social support systems and cultural backgrounds may limit the generalizability of the results and contribute to heterogeneity.

This study also has certain limitations. First, the absence of a systematic evaluation of the evidence certainty (e.g., using the Grading of Recommendations Assessment, Development and Evaluation framework) is a notable methodological limitation. This omission means that readers and clinicians should exercise caution in applying these findings, as the true effect might be substantially different. Furthermore, the literature included in this research was limited to Chinese publications, representing a constraint in the scope of included studies. Due to factors such as differences in sample sources and characteristics, variations in specific intervention measures, the lack of blinding, and assessment methods, the quality of the included literature was rated as moderate. Among the included studies, the primary racial group of the research subjects was of Asian descent (Yellow race), while some studies also included other races. This may have contributed to increased heterogeneity. Therefore, the effectiveness of psychological interventions on anxiety and depression in cancer pain patients requires further investigation. Future research should conduct more rigorous, large-sample, multicenter RCTs with more refined classifications to examine the effects of different psychological interventions on anxiety, depression, and other adverse emotions in patients with various types of cancer pain and from different racial groups.

## 5. Conclusion

Psychological intervention can effectively improve the anxiety and depression of cancer patients during chemotherapy, and future studies need to pay attention to the impact of factors such as the type of cancer, cancer pain, patient population, and specific psychological interventions.

## Author contributions

**Conceptualization:** Xiao Pu Wu, Jin Liang Kong, Jing Luo.

**Methodology:** Jin Ling Tang, Cao Qing.

**Data curation:** Yi Wang, Shi Hao Yang, Chong Xi Bao, Qin Zhe Zhang.

**Formal analysis:** Yi Wang, Shi Hao Yang, Chong Xi Bao, Qin Zhe Zhang.

**Software:** Shang Li, Yun Jiang, Wei Lu.

**Visualization:** Shang Li, Yun Jiang, Wei Lu.

**Investigation:** Jin Ling Tang, Cao Qing.

**Project administration:** Xiao Pu Wu, Jin Liang Kong, Jing Luo.

**Resources:** Xiao Pu Wu, Shao Chu Zheng, Yu Ting Huang.

**Supervision:** Xiao Pu Wu, Jin Liang Kong, Jing Luo.

**Validation:** Xiao Pu Wu, Jin Liang Kong, Jing Luo.

**Writing – original draft:** Xiao Pu Wu, Yue Wu.

**Writing – review & editing:** Xiao Pu Wu, Jing Luo.


