# Supplementary material for: The impact of psychological interventions on anxiety and depression in cancer pain patients: A meta-analysis
Source: Medicine (Baltimore). 2026 May 8;105(19):e48585. doi: 10.1097/MD.0000000000048585 (PMC13166464; doi:10.1097/MD.0000000000048585)
Supplement: Supplementary file 1 [file medi-105-e48585-s001.docx]

**CNKI (China National Knowledge Infrastructure) Database Search Strategy:**

(SU=('cancer pain' + 'cancer pain patients' + 'cancer pain patients')) OR (SU=('cancerous pain' + 'cancerous pain patients')) AND (SU=('anxiety' + 'anxiety and depression' + 'anxiety emotion' + 'anxiety state')) OR (SU=('depression' + 'depressive symptoms' + 'depressed patients')) AND (SU=('psychological intervention' + 'psychological intervention therapy' + 'psychological intervention methods')) OR (SU=('cognitive behavioral therapy' + 'cognitive behavioral therapy intervention')) OR (SU=('supportive psychotherapy')) OR (SU=('mindfulness therapy')) OR (SU=('time perspective therapy')) AND (KFY=('random' [Exact]) OR (KFY=('randomized controlled' + 'randomized controlled trial (rct)' + 'randomized controlled experiment' + 'randomized controlled method' [Exact]))

**Wanfang Database Search Strategy:**

Search Expression: SU:('cancerous pain') AND SU:('adverse psychological emotions' OR 'negative emotions' OR 'anxiety' OR 'fear' OR 'depression' OR 'anger' OR 'tension' OR 'despair') AND SU:('psychological intervention therapy' OR 'psychological intervention measures' OR 'psychological intervention methods' OR 'psychological intervention') AND SU:('randomized controlled' OR 'random allocation' OR 'random')

**CBM (SinoMed) Database Search Strategy:**

((("randomized controlled"[Common Fields: Intelligent] OR "random"[Common Fields: Intelligent] OR "RCT"[Common Fields: Intelligent]) OR ("randomized controlled trial"[Unweighted: Extended])) AND (((("psychological intervention therapy"[Common Fields: Intelligent] OR "psychological intervention measures"[Common Fields: Intelligent] OR "psychological intervention methods"[Common Fields: Intelligent] OR "psychological intervention"[Common Fields: Intelligent]) OR ("psychosocial intervention"[Unweighted: Extended])) AND (((("negative emotions"[Common Fields: Intelligent]) OR ("adverse emotions"[Common Fields: Intelligent]) OR ("anxiety"[Common Fields: Intelligent]) OR ("tension"[Unweighted: Extended]) OR ("anger management therapy"[Unweighted: Extended]) OR ("fear"[Unweighted: Extended]) OR ("depression"[Unweighted: Extended]) OR ("depressive"[Unweighted: Extended]) OR ("panic disorder"[Unweighted: Extended])) AND (("neoplastic pain"[Common Fields: Intelligent]) OR ("cancer pain"[Common Fields: Intelligent]) OR ("cancerous pain patients"[Common Fields: Intelligent]) OR ("cancerous pain"[Unweighted: Extended]))))

**VIP Database Search Strategy:**

(((((((TI=('cancer pain') OR KY=('cancer pain')) OR TI=('cancerous pain')) OR TI=('cancer pain')) OR TI=('neoplastic pain')) AND (((((((TI=('negative emotions') OR KY=('negative emotions')) OR TI=('adverse emotions') OR KY=('adverse emotions')) OR TI=('anger') OR KY=('anger')) OR TI=('anxiety') OR KY=('anxiety')) OR TI=('depression') OR KY=('depression')) OR TI=('stigma') OR KY=('stigma')) OR TI=('tension') OR KY=('tension')) OR TI=('despair') OR KY=('despair'))) AND ((((TI=('psychological intervention therapy') OR KY=('psychological intervention therapy')) OR TI=('psychological intervention') OR KY=('psychological intervention')) OR TI=('psychological intervention measures') OR KY=('psychological intervention measures')) OR TI=('psychological intervention methods') OR KY=('psychological intervention methods')) OR TI=('psychological intervention therapy') OR KY=('psychological intervention therapy'))) AND ((((AB=('RCT') OR AB=('randomized controlled')) OR AB=('random')) OR AB=('random allocation')))

**PubMed Database Search Strategy:**

((("Cancer Pain"[Mesh]) OR ((((((((((((((((((((((((((((((((((((((((Pain, Cancer[Title/Abstract]) OR (Pains, Cancer[Title/Abstract])) OR (Cancer-Related Pain[Title/Abstract])) OR (Cancer Related Pain[Title/Abstract])) OR (Cancer-Related Pains[Title/Abstract])) OR (Pain, Cancer-Related[Title/Abstract])) OR (Pains, Cancer-Related[Title/Abstract])) OR (Cancer-Associated Pain[Title/Abstract])) OR (Cancer Associated Pain[Title/Abstract])) OR (Cancer-Associated Pains[Title/Abstract])) OR (Pain, Cancer-Associated[Title/Abstract])) OR (Pains, Cancer-Associated[Title/Abstract])) OR (Neoplasm-Related Pain[Title/Abstract])) OR (Neoplasm Related Pain[Title/Abstract])) OR (Neoplasm-Related Pains[Title/Abstract])) OR (Pain, Neoplasm-Related[Title/Abstract])) OR (Pains, Neoplasm-Related[Title/Abstract])) OR (Neoplasm-Associated Pain[Title/Abstract])) OR (Neoplasm Associated Pain[Title/Abstract])) OR (Neoplasm-Associated Pains[Title/Abstract])) OR (Pain, Neoplasm-Associated[Title/Abstract])) OR (Pains, Neoplasm-Associated[Title/Abstract])) OR (Oncological Pain[Title/Abstract])) OR (Oncological Pains[Title/Abstract])) OR (Pain, Oncological[Title/Abstract])) OR (Pains, Oncological[Title/Abstract])) OR (Tumor-Related Pain[Title/Abstract])) OR (Pains, Tumor-Related[Title/Abstract])) OR (Pain, Tumor-Related[Title/Abstract])) OR (Tumor Related Pain[Title/Abstract])) OR (Tumor-Related Pains[Title/Abstract])) OR (Tumor-Associated Pain[Title/Abstract])) OR (Pains, Tumor-Associated[Title/Abstract])) OR (Pain, Tumor-Associated[Title/Abstract])) OR (Tumor Associated Pain[Title/Abstract])) OR (Tumor-Associated Pains[Title/Abstract])) OR (Oncology Pain[Title/Abstract])) OR (Oncology Pains[Title/Abstract])) OR (Pain, Oncology[Title/Abstract])) OR (Pains, Oncology[Title/Abstract]))) AND ((((((("Fear"[Mesh]) OR ((((((Threat Cues[Title/Abstract]) OR (Cue, Threat[Title/Abstract])) OR (Threat Cue[Title/Abstract])) OR (Threat Sensitivity[Title/Abstract])) OR (Sensitivity, Threat[Title/Abstract])) OR (Threat Sensitivities[Title/Abstract]))) OR (("Anger"[Mesh]) OR (angers[Title/Abstract]))) OR (("Anxiety"[Mesh]) OR ((((((((Angst[Title/Abstract]) OR (Nervousness[Title/Abstract])) OR (Hypervigilance[Title/Abstract])) OR (Social Anxiety[Title/Abstract])) OR (Anxieties, Social[Title/Abstract])) OR (Anxiety, Social[Title/Abstract])) OR (Social Anxieties[Title/Abstract])) OR (Anxiousness[Title/Abstract])))) OR (("Depression"[Mesh]) OR (((((Depressive Symptoms[Title/Abstract]) OR (Depressive Symptom[Title/Abstract])) OR (Symptom, Depressive[Title/Abstract])) OR (Emotional Depression[Title/Abstract])) OR (Depression, Emotional[Title/Abstract])))) OR (((despair[Title/Abstract]) OR (stigma[Title/Abstract])) OR (nervous[Title/Abstract]))) OR ((negative emotion[Title/Abstract]) OR (unhealthy emotions[Title/Abstract])))) AND ((("Psychosocial Intervention"[Mesh]) OR (((((((Intervention, Psychosocial[Title/Abstract]) OR (Interventions, Psychosocial[Title/Abstract])) OR (Psychosocial Interventions[Title/Abstract])) OR (Psychological Intervention[Title/Abstract])) OR (Intervention, Psychological[Title/Abstract])) OR (Interventions, Psychological[Title/Abstract])) OR (Psychological Interventions[Title/Abstract]))) OR (Psychotherapy[Title/Abstract]))) AND (randomized controlled trial[Publication Type] OR randomized[Title/Abstract] OR placebo[Title/Abstract])

**Web of Science (WOS) Database Search Strategy:**

(TS=("cancer pain" OR "oncologic pain" OR "neoplas* pain" OR "malignancy-related pain")) AND (TS=("negative emotion*" OR "psychological distress" OR anxiety OR depress* OR fear OR anger)) AND (TS=("psychological intervention*" OR "psychosocial intervention*" OR psychotherap* OR "cognitive behavioral therapy" OR "supportive psychotherapy" OR "mindfulness-based intervention*")) AND (AB=(random* OR RCT OR "randomized controlled trial" OR placebo))

**Cochrane Library Database Search Strategy:**

#1 MeSH descriptor: [Cancer Pain] explode all trees

#2 (Cancer Pains):ti,ab,kw OR (Pain, Cancer):ti,ab,kw OR (Pains, Cancer):ti,ab,kw OR (Cancer-Related Pain):ti,ab,kw OR (Cancer Related Pain):ti,ab,kw OR (Cancer-Related Pains):ti,ab,kw OR (Pain, Cancer-Related):ti,ab,kw OR (Pains, Cancer-Related):ti,ab,kw OR (Cancer-Associated Pain):ti,ab,kw OR (Cancer Associated Pain):ti,ab,kw OR (Cancer-Associated Pains):ti,ab,kw OR (Pain, Cancer-Associated):ti,ab,kw OR (Pains, Cancer-Associated):ti,ab,kw OR (Neoplasm-Related Pain):ti,ab,kw OR (Neoplasm Related Pain):ti,ab,kw OR (Neoplasm-Related Pains):ti,ab,kw OR (Pain, Neoplasm-Related):ti,ab,kw OR (Neoplasm-Associated Pain):ti,ab,kw OR (Neoplasm Associated Pain):ti,ab,kw OR (Neoplasm-Associated Pains):ti,ab,kw OR (Pain, Neoplasm-Associated):ti,ab,kw OR (Pains, Neoplasm-Associated):ti,ab,kw OR (Oncological Pain):ti,ab,kw OR (Oncological Pains):ti,ab,kw OR (Pain, Oncological):ti,ab,kw OR (Pains, Oncological):ti,ab,kw OR (Tumor-Related Pain):ti,ab,kw OR (Pains, Tumor-Related):ti,ab,kw OR (Pain, Tumor-Related):ti,ab,kw OR (Tumor Related Pain):ti,ab,kw OR (Tumor-Related Pains):ti,ab,kw OR (Tumor-Associated Pain):ti,ab,kw OR (Pains, Tumor-Associated):ti,ab,kw OR (Pain, Tumor-Associated):ti,ab,kw OR (Tumor Associated Pain):ti,ab,kw OR (Tumor-Associated Pains):ti,ab,kw OR (Oncology Pain):ti,ab,kw OR (Oncology Pains):ti,ab,kw OR (Pain, Oncology):ti,ab,kw OR (Pains, Oncology):ti,ab,kw

#3 #1 OR #2

#4 (Negative emotions):ti,ab,kw

#5 MeSH descriptor: [Psychosocial Intervention] explode all trees

#6 (Intervention, Psychosocial):ti,ab,kw OR (Interventions, Psychosocial):ti,ab,kw OR (Psychosocial Interventions):ti,ab,kw OR (Psychological Intervention):ti,ab,kw OR (Intervention, Psychological):ti,ab,kw OR (Interventions, Psychological):ti,ab,kw OR (Psychological Interventions):ti,ab,kw OR (Psychotherapy):ti,ab,kw

#7 #5 OR #6

#8 #3 and #4

#9 #7 and #8

**Embase Database Search Strategy:**

#22 #18 AND #19 AND #21

#21 #3 AND #20

#20 #6 OR #9 OR #12 OR #15

#19 'randomized controlled trial':ab,ti OR 'randomized':ab,ti OR 'placebo':ab,ti

#18 #16 OR #17

#17 'intervention, psychosocial':ab,ti OR 'interventions, psychosocial':ab,ti OR 'psychosocial interventions':ab,ti OR 'psychological intervention':ab,ti OR 'intervention, psychological':ab,ti OR 'interventions, psychological':ab,ti OR 'psychological interventions':ab,ti OR 'psychotherapy':ab,ti

#16 'psychosocial intervention'/exp OR 'psychosocial intervention'

#15 #13 OR #14

#14 'depressive symptoms':ab,ti OR 'depressive symptom':ab,ti OR 'symptom, depressive':ab,ti OR 'emotional depression':ab,ti OR 'depression, emotional':ab,ti

#13 'depression'/exp OR 'depression'

#12 #10 OR #11

#11 'angst':ab,ti OR 'nervousness':ab,ti OR 'hypervigilance':ab,ti OR 'social anxiety':ab,ti OR 'anxieties, social':ab,ti OR 'anxiety, social':ab,ti OR 'social anxieties':ab,ti OR 'anxiousness':ab,ti

#10 'anxiety'/exp OR 'anxiety'

#9 #7 OR #8

#8 'angers':ab,ti

#7 'anger'/exp OR 'anger'

#6 #4 OR #5

#5 'fears':ab,ti OR 'threat cues':ab,ti OR 'cue, threat':ab,ti OR 'threat cue':ab,ti OR 'threat sensitivity':ab,ti OR 'sensitivity, threat':ab,ti OR 'threat sensitivities':ab,ti

#4 'fear'/exp OR 'fear'

#3 #1 OR #2

#2 'cancer pains':ab,ti OR 'pain, cancer':ab,ti OR 'pains, cancer':ab,ti OR 'cancer-related pain':ab,ti OR 'cancer related pain':ab,ti OR 'cancer-related pains':ab,ti OR 'pain, cancer-related':ab,ti OR 'pains, cancer-related':ab,ti OR 'cancer-associated pain':ab,ti OR 'cancer associated pain':ab,ti OR 'cancer-associated pains':ab,ti OR 'pain, cancer-associated':ab,ti OR 'pains, cancer-associated':ab,ti OR 'neoplasm-related pain':ab,ti OR 'neoplasm related pain':ab,ti OR 'neoplasm-related pains':ab,ti OR 'pain, neoplasm-related':ab,ti OR 'pains, neoplasm-related':ab,ti OR 'neoplasm-associated pain':ab,ti OR 'neoplasm associated pain':ab,ti OR 'neoplasm-associated pains':ab,ti OR 'pain, neoplasm-associated':ab,ti OR 'pains, neoplasm-associated':ab,ti OR 'oncological pain':ab,ti OR 'oncological pains':ab,ti OR 'pain, oncological':ab,ti OR 'pains, oncological':ab,ti OR 'tumor-related pain':ab,ti OR 'pains, tumor-related':ab,ti OR 'pain, tumor-related':ab,ti OR 'tumor related pain':ab,ti OR 'tumor-related pains':ab,ti OR 'tumor-associated pain':ab,ti OR 'pains, tumor-associated':ab,ti OR 'pain, tumor-associated':ab,ti OR 'tumor associated pain':ab,ti OR 'tumor-associated pains':ab,ti OR 'oncology pain':ab,ti OR 'oncology pains':ab,ti OR 'pain, oncology':ab,ti OR 'pains, oncology':ab,ti

#1 'cancer pain'/exp OR 'cancer pain'
